# Targeting the neonatal Fc receptor (FcRn) is not beneficial in an animal model of chronic neuritis

**DOI:** 10.1007/s12026-024-09565-7

**Published:** 2024-12-15

**Authors:** Anne K. Mausberg, Fabian Szepanowski, Bianca Eggert, Kai C. Liebig, Christoph Kleinschnitz, Bernd C. Kieseier, Mark Stettner

**Affiliations:** 1 Department of Neurology, Center for Translational Neuro- and Behavioural Sciences, University Hospital Essen, Hufelandstr. 55, 45147 Essen, Germany; 2 Department of Neurology, University of Duesseldorf, Moorenstr. 5, 40225 Düsseldorf, Germany

**Keywords:** ICAM-deficient NOD mouse, Chronic inflammatory demyelinating polyradiculoneuropathy (CIDP), Intravenous immunoglobulins (IVIg), Immune-mediated neuropathy, Neonatal Fc receptor (FcRn)

## Abstract

The inhibition of the neonatal Fc receptor (FcRn) is a promising therapeutic pathway in certain autoimmune disorders to reduce the amount of circulating pathogenic IgG autoantibodies by interfering with their recycling system. FcRn antibodies are currently being tested in chronic inflammatory demyelinating polyradiculoneuropathy (CIDP). This study aimed to investigate the therapeutic potential of an antibody targeting FcRn in the intracellular adhesion molecule 1 (ICAM1)-deficient NOD mouse—a model representative for many aspects of human CIDP. After the onset of clinical signs of neuropathy, ICAM1-deficient NOD mice were assigned to treatment twice per week with anti-FcRn antibody, isotype control antibody (negative control) or intraperitoneal (administered) immunoglobulin (positive control). Disease severity was monitored using disease-specific assessments for ataxia and paresis such as grip strength measurements. Serum immunoglobulin levels and peripheral nerve immune cell infiltration were quantified. Treatment with anti-FcRn antibody did not ameliorate disease progression, as determined by clinical scores and grip strength analysis. Disease progression was reduced in the positive control animals receiving immunoglobulin. Consistent with the clinical results, the composition of infiltrating immune cells was not altered in the peripheral nerve of anti-FcRn antibody-treated mice compared to controls. However, in anti-FcRn antibody-treated mice, significantly lower IgG levels were detectable compared to controls. These findings suggest that targeting the FcRn recycling system does not influence disease progression in the NOD-ICAM1-deficient mouse model of CIDP. Further studies will elucidate whether the reduction of IgG levels was insufficient to deplete pathogenic autoantibodies or whether the major inflammatory driver in the NOD-ICAM1-deficient mouse animal model is mediated by factors other than pathological immunoglobulins.

## Background

In chronic autoimmune disorders of the peripheral nervous system, an activation of humoral as well as cellular immune responses is known to contribute to disease progression [1]. The therapeutic options for this group of diseases are limited to pleiotropic immunomodulatory and immunosuppressive drugs—immunoglobulins or corticosteroids being the most commonly used [2]. About 70–80% of patients with chronic inflammatory demyelinating polyradiculoneuropathy (CIDP) show clinical response to intravenous immunoglobulin (IVIg) treatment [3]; however, as a product derived from human plasma, its supply is limited. IVIg addresses multiple targets in the immune system, attenuating complement activity, reducing the number of activated T cells and dampening phagocytic activity of macrophages [4, 5]. In addition, it is hypothesised that, beyond these pleiotropic effects, a dilution of pathological autoantibodies and reduction in the accessibility of autoantibodies to their specific target may ameliorate disease activity in CIDP.

A more specific approach to reducing the number of autoantibodies is to target the neonatal Fc receptor (FcRn)—the central regulator of the endogenous IgG recycling machinery. Usually, FcRn protects bound antibodies from degradation, thereby increasing the half-life and bioavailability of circulating immunoglobulins [6]. Blocking this receptor leads to the degradation of antibodies and results in a reduced number of antibodies in general, including detrimental self-reactive autoantibodies [7]. The pronounced reduction of serum immunoglobulins by FcRn modulation is a novel emerging therapeutic target, which is currently under investigation for CIDP, with very promising preliminary results of clinical studies.

This study aimed to evaluate an FcRn blocking antibody in an established animal model of chronic neuritis, the NOD-ICAM1-deficient mouse. This animal model of chronic neuritis shares relevant aspects of the cellular and humoral immunopathology with CIDP [8]. We have previously confirmed the effectiveness of intraperitoneal treatment with a human IVIg formulation in the NOD-ICAM1-deficient model [9]; therefore, this study set out to compare FcRn blockade versus a control antibody or established treatment with a human intraperitoneal (administered) immunoglobulin (hipIg).

## Methods

### Approvals and registrations

Animal experimentation was approved by the responsible state authorities (Landesamt für Natur, Umwelt und Verbraucherschutz Nordrhein-Westfalen) under the approval reference number 84–02.04.2015.A108.

### HipIg and anti-FcRn treatment of ICAM-deficient NOD mice

At 1 year of age, female ICAM1^tm1Jcgr^NOD mice develop a spontaneous autoimmune peripheral neuritis, as previously described [8]. At 12 to 18 months of age, female ICAM 1^tm1Jcgr^NOD mice were treated for a period of 7 weeks, twice per week, with either 100 µl 10% human IVIg (Baxter), anti-FcRn antibody 4470 (murinised IgG1 anti-mouse FcRn, 30 mg/kg bodyweight) or unspecific isotype control antibody 101.4 (an unspecific mouse IgG1 anti-human TNF antibody, mouse IgG1, 30 mg/kg bodyweight), all administered intraperitoneally. Animals were pooled from two independent experiments, *n* = 15 per group. Mice were examined daily. Twice weekly, a modified clinical score for paresis and ataxia was assessed to monitor for clinical signs of neuropathy. Paresis score was assessed using the limb clasping test according to the following scale: score of 0, limbs splayed outwards to keep balance; 1–4, limbs are continuously retracted (each limb separately counted); summed 5, severe motor impairments; and 6, death due to neuropathy. Ataxia score was attained from a walking test to assess skilled walking and measure both forelimbs and hindlimbs. Slight footfaults were scored with 1, severe ones scored 2 for every limb, summed score 9 for moribund and 10 for death due to neuropathy. After 7 weeks of treatment, blood was collected and serum was centrifuged. Mice were sacrificed and perfused first with phosphate-buffered saline (PBS) and then with paraformaldehyde.

### Grip strength analysis

Once per week, grip strength analysis of the hindlimbs using a modified force gauge (Erichsen Physimeter 906 MC-B) was conducted. The mouse was handled by the experimenter with a tight grip behind its head, while still allowing the hind limbs to move freely. Subsequently, the hind limbs were gently pulled over a metal bar, and the maximum force applied before the mouse lost its grip was recorded. Mice were tested three times in succession, and data were averaged for each mouse and each time point.

### Immunohistology

Organs were embedded in Tissue-Tek (Sakura, Netherlands) and frozen at − 80 °C. Ten-micrometre sections (Cryostat CM1950 Leica, Wetzlar, Germany) were stained with the following antibodies: rabbit anti-mouse CD3 (mAB #ab16669, Abcam), rat anti-mouse CD45R/B220 (mAB #MCA1258G, Biorad) and the corresponding fluorescent conjugated secondary antibodies (goat anti-rat AF594, goat anti-rabbit AF488, Abcam) and 4,6′diamidino-2-phenylindole (DAPI, Sigma). Slices were covered using Mowiol/Dabco (Roth) mounting medium, and images were photographed with a DMi8 (Leica) microscope.

### Measurement of IgM and IgG levels in serum

Serum was collected 7 weeks after treatment initiation in the three groups. Mouse IgM and mouse IgG enzyme-linked immunosorbent assays (ELISA) were performed according to the manufacturer’s protocol (Abcam). This mouse ELISA kit excluded the detection of human IgG and IgM to avoid cross-reactivity with human IVIg. Serum, as well as standard curve absorption, was measured in triplicate on an Infinite M200 Pro (Tecan, Germany) and the i-control™ software.

### Data analysis

GraphPad Prism version 7.0 (GraphPad Software, San Diego, CA) was used for statistical analysis. The Mann–Whitney test was used to assess for statistically significant differences in clinical score values. Student’s *t*-test for unrelated samples was used to investigate statistically significant differences in all other analyses. Differences were considered significant at *p* values ≤ 0.05.

## Results

### Disease progression is not ameliorated by anti-FcRn antibody treatment

**Fig. 1:**
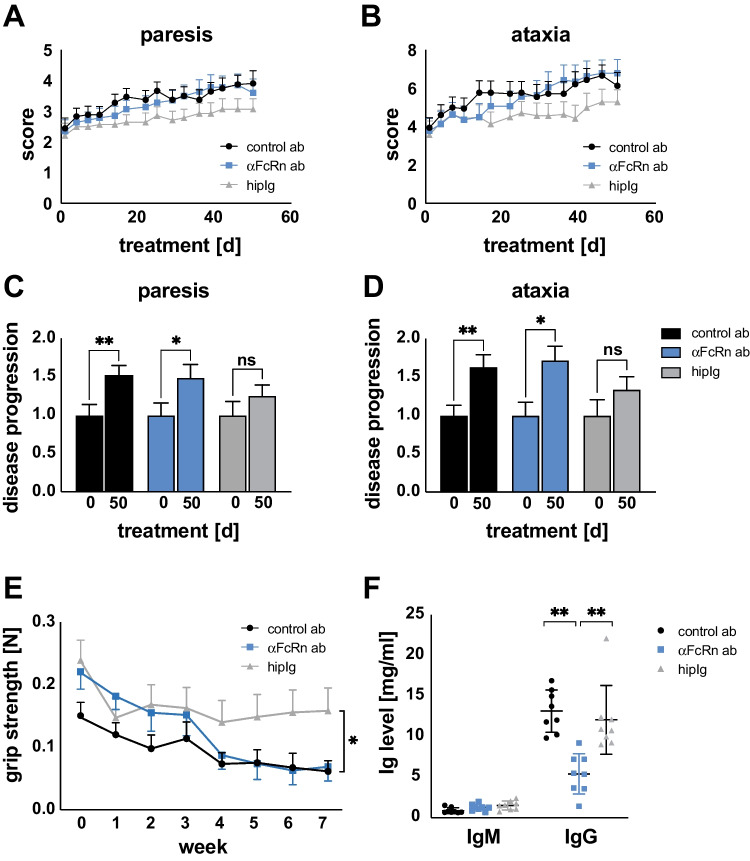
Treatment with anti-FcRn antibody does not ameliorate disease progression. Clinical evaluation of diseased ICAM1-deficient NOD mice treated with anti-FcRn antibody (blue), isotype control antibody (black) or hipIg (grey) for 7 weeks. **A** Paresis score: limb clasping test: score of 0: limps splayed outwards to keep balance; 1: limps are continuously retracted (every limp separately counted); summed 5: severe motor impairments; 6: death due to neuropathy. **B** Ataxia score: walking test to assess skilled walking and measure both forelimbs and hindlimbs. Slight foot faults were scored with 1; severe ones scored 2 for every limb; summed score 9: moribund; 10: death due to neuropathy. Depicted is mean ± SEM. Delta score of **C** paresis and **D** ataxia between the start and endpoint of treatment was determined to illustrate disease progression. For calculation, scores from endpoint were divided by the scores at day 0. Depicted is mean ± SEM (asterisks indicate significance: *p < 0.05; **p < 0.01; unpaired Student’s *t*-test). **E** Grip strength (force in Newton) analysis of diseased ICAM1-deficient NOD mice treated for 50 days with anti-FcRn antibody (blue), isotype control antibody (black) or hipIg (grey). Three independent measurements per animal were averaged. Depicted is mean ± SEM (asterisks indicate significance: *p ≤ 0.05; Mann–Whitney test). **F** After 50 days of treatment, serum levels of mouse IgM and IgG were determined using ELISA. Depicted is mean ± SEM (asterisks indicate significance: **p < 0.01; unpaired Student’s *t*-test)

Previous studies established ICAM1-deficient NOD mice as an animal model representative for many aspects of CIDP and demonstrated the responsiveness of this model to immunoglobulin [8], [9]. Thus, hipIg was employed as a positive control in this assessment of anti-FcRn receptor blockade. An unspecific isotype antibody was used as a negative control. A cohort of animals (*n* = 50; 15–20 in each group) with moderate symptoms (mean clinical paresis score 2.34) was used in this study. The animal model is characterised by slow chronic progression with both clinical scores—the paresis score to measure muscle weakness and the ataxia score to address sensorimotor coordination—continuously increasing as disease worsens (Fig. 1).

In the negative control group, the paresis score steadily increased over time, indicating deterioration of clinical disease indicators. This worsening of the condition was not altered by anti-FcRn antibody treatment (Fig. 1A). In contrast, treatment with hipIg treatment slowed disease progression, as indicated by the paresis score (Fig. 1A). Similar results were obtained for sensorimotor function using the ataxia score (Fig. 1B). Anti-FcRn treatment did not change the disease course compared to control antibody treatment, while hipIg-treated animal showed more stable ataxia scores over time. To analyse disease progression in detail, we calculated delta scores from the start (day 0) to the endpoint (day 50) of treatment (Fig. 1C). Anti-FcRn antibody-treated animals showed a significant increase in the paresis score between day 0 and day 50 following treatment, comparable to isotype controls. Animals treated with hipIg showed no significant difference in the delta score between the two time points, indicative of no significant change in the paresis score. Similar findings were obtained following analysis of the change in ataxia score over time (Fig. 1D). Taken together, these results show that anti-FcRn antibody treatment is not effective in this mouse model of chronic neuritis, while hipIg treatment slowed disease progression. We further analysed the grip strength of ICAM-deficient NOD mice to determine treatment efficacy (Fig. 1E). In the hipIg-treated group, grip strength did not show a deterioration over time, while it declined progressively in both the anti-FcRn antibody-treated group and isotype negative control group. These findings indicate that, while hipIg treatment stabilised disease progression and enabled animals to maintain grip strength, anti-FcRn antibody treatment was not effective.

### Anti-FcRn antibody treatment reduces IgG levels

To determine the effectiveness of the antibodies to block the FcRn recycling system, serum IgM and IgG levels were measured by ELISA 50 days following treatment of ICAM-deficient NOD mice with anti-FcRn antibody, isotype control or hipIg (Fig. 1F). Anti-FcRn antibody treatment induced a serum IgG reduction of approximately 65% compared to serum levels in the isotype control and hipIg groups, indicating that blocking the FcRn recycling system effectively reduces the concentration of serum IgG. IgG levels of hipIg and control antibody-treated animals did not differ. As expected, serum levels of IgM were not affected by treatment with the anti-FcRn antibody or by hipIg treatment.

### Peripheral nerve inflammation is not affected following anti-FcRn antibody treatment

**Fig. 2:**
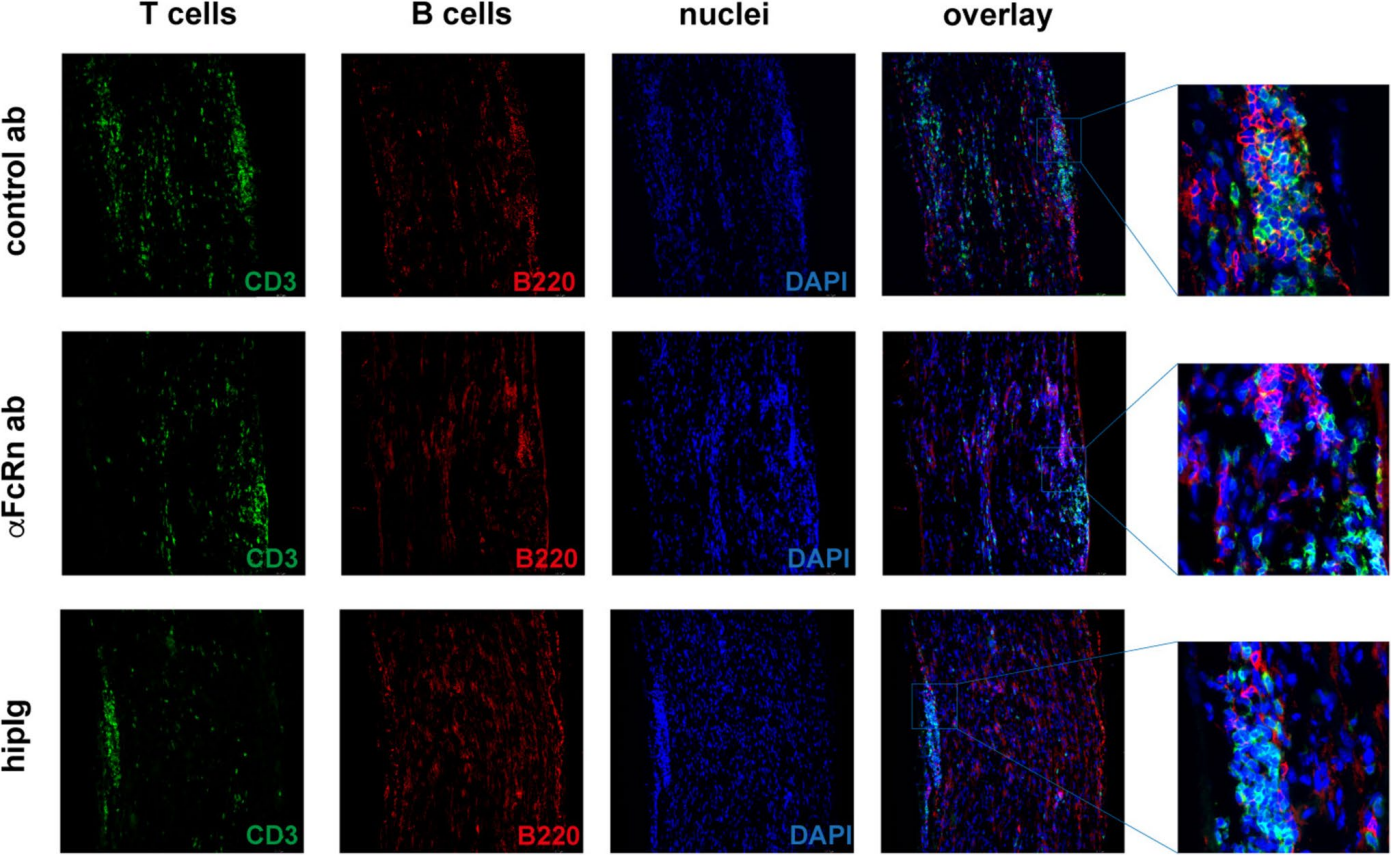
Treatment with anti-FcRn antibody did not affect nerve cell infiltration. Immunohistochemical staining of sciatic nerves of affected ICAM1-deficient NOD mice treated for 50 days with anti-FcRn antibody, unspecific control antibody or hipIg. T cells (CD3, green), B cells (B220, red) and cell nuclei (DAPI, blue) staining revealed no major differences in nerve cell infiltration or composition between the three treatment groups. Representative images are shown

Immunohistochemical staining of sciatic nerves enabled the examination of nerve cell infiltration in longitudinal sections (Fig. 2). T cells (CD3, green), B cells (B220, red) and cell nuclei (DAPI, blue) were stained. T cells infiltrated the peripheral nerve in a patchy pattern, while B cells were also found outside of the bulked accumulation. Infiltrates were distributed evenly throughout the nerve with no distal or proximal preference. Regardless of the treatment regimen, the composition of the infiltrates was comparable and contained patchy accumulations of T cells and B cells (see also higher magnification). As previously described, hipIg-treated animals showed reduced infiltrations [9]. We also counted T cells and B cells in five randomly selected fields in three sections of three representative animals per group but could detect no significant differences in T cell or B cell numbers or in the ratio of both (data not shown). Taken together, the three treatment regimens only had a minor effect on the composition of cellular infiltrates.

## Discussion

This study demonstrates that reduction of IgG levels by targeting the FcRn recycling system does not mitigate peripheral nerve inflammation in ICAM1-deficient NOD mice—an animal model for chronic neuritis.

Targeting the FcRn mechanism has been established as a new therapeutic approach for the treatment of IgG-mediated autoimmune diseases. Clinical trials to study FcRn blockade have been initiated for various conditions such as immune thrombocytopenia (ITP-phase 2 trial) [10, 11], generalised myasthenia gravis (MG-phase 2 trials [12, 13], MG-phase 3 trials [14, 15]) and CIDP. In immune-mediated neuropathies, FcRn modulation is a promising candidate and potential alternative to IVIg, corticosteroids or apheresis therapy.

It is anticipated that FcRn blockade might be beneficial for a subgroup of CIDP patients, targeting pathophysiologically relevant IgGs and enabling a more individually tailored treatment strategy. A recent study in CIDP patients tested an FcRn monoclonal antibody, (IgG4P) rozanolixizumab, which led to > 80% reduction of IgG but failed to meet the primary endpoint, which was changed from baseline to week 13 in inflammatory Rasch-built Overall Disability Scale (iRODS) score [16]. Comparison of treatment efficacy was complicated because almost two-thirds of patients in the placebo arm remained stable, possibly due to the study design, which only included patients with historic IVIg dependency. It is hoped that ongoing, well-designed clinical trials in CIDP patients may provide further insights on the potential for FcRn modulating agents in this complex condition. Ongoing phase 2 trials in patients with CIDP with batoclimab (NCT05581199) and nipocalimab (NCT05327114)—agents targeting the FcRn system—will explore the value of this therapeutic approach. Recently, a clinical trial treating patients with CIDP with efgartigimod (ADHERE + ; NCT04280718) showed promising efficacy.

Based on these results, the FDA most recently approved efgartigimod as a new treatment for patients with CIDP. The therapeutic approach does work, at least in a subset of CIDP patients since the study analysed a highly enriched cohort of CIDP patients in terms of stratification. However, in the best-case scenario, the efficacy of efgartigimod addressing the FcRn system may be partially based on complementary mechanisms beyond autoantibody reduction, such as modulation of macrophages.

In our animal study, an anti-mouse monoclonal FcRn antibody of the IgG1 isotype has been employed, and treatment resulted in a reduction of serum IgG levels of 65%, while IgM levels were not affected. Conceivably, this IgG reduction may not have been strong enough to induce a clinical treatment effect in these NOD-ICAM1-deficient mice. Moreover, the importance of B cells and the humoral immune system in the NOD-ICAM-deficient mouse model is still a matter of debate. Despite the fact that adoptive transfer of T cells can induce the disease, the humoral immune system also seems to be involved in its pathophysiology since nerve infiltrates do not only comprise T cells but also contain high numbers of B cells [8]. Indeed, in single-cell transcriptomics of the NOD-ICAM1-deficient mouse, nerve infiltrates contained B cells with an activated phenotype [17]. The serum of affected animals reacts with different components of the PNS, indicating the presence of self-reacting antibodies in the NOD-ICAM-deficient mouse model [8]. Nevertheless, the destructive capacity of these autoantibodies is still not proven, and it is not clear to what extent they result in disease. Our data indicate that reduction of IgG might not ameliorate autoimmune neuritis in the NOD-ICAM1-deficient mouse. Rather, the data suggest that the therapeutic effect of hipIg—at least in the neuritis mouse model—is independent on IgG levels, pleiotropic and not only driven by their ability to reduce IgG recycling by FcRn saturation. This may also be relevant for a subgroup of patients with CIDP. Here, it has been speculated that the initial immune response is T cell driven, while chronic disease phase is underpinned by predominantly humoral responses [18]. Ongoing inflammatory responses could induce the process of epitope spreading, thereby broadening the antigenic repertoire, leading to a secondary relevance of autoantibodies. Although T cell proliferation [19] and increased numbers of CD4^+^ T cells and CD8^+^ T cells featuring oligoclonal expansions in patients with CIDP [20, 21] underline the importance of cytotoxic T cells in the pathogenesis of CIDP [20, 21], antibodies against structures of the peripheral nervous system have been recently described [22]. These findings may define new subtypes of immune-mediated neuropathies and further emphasise the role of the humoral immune response in CIDP in the coming years.

The broad efficacy of IVIg in the treatment of CIDP is rooted in the ability to exert a number of different mechanisms of action [4] such as the reduction in oligoclonal T cell expansion, particularly within the CD8^+^ T cell population [20]. IVIg treatment is also associated with an alteration of regulatory NK cell numbers [23] in CIDP patients. Since a proportion of patients do not respond to IVIg, corticosteroids or apheresis therapy, and IVIg availability remains a problem, there is an unmet need to broaden the therapeutic armamentarium in immune-mediated neuropathies. FcRn blockade might be a suitable option for a group of CIDP patients, targeting pathophysiologically relevant IgGs and enabling more individually tailored treatment strategies.

## Conclusions

Anti-FcRn antibody treatment reduced the concentration of serum IgGs in ICAM1-deficient NOD mice—a model for chronic neuritis—but this effect did not translate into an amelioration of clinical symptoms or attenuation of histological inflammation. Pathophysiological mechanisms in this model may not be primarily driven by autoantibodies or, perhaps, the reduction of IgG levels was insufficient to deplete detrimental autoantibodies. Ongoing studies will help to elucidate FcRn as a promising target in the treatment of immune-mediated neuropathies.

## Data Availability

All data supporting the conclusion of this study are included in this article, further inquiries can be directed to the corresponding author.

## Abbreviations

CIDP: Chronic inflammatory demyelinating polyradiculoneuropathy
FcRn: Neonatal Fc receptor
HipIg: Human intraperitoneal (administered) immunoglobulin
ITP: Immune thrombocytopenia
IVIg: Intravenous immunoglobulins
NK: Natural killer (cells)
